# The establishment, maintenance, and adaptation of high- and low-impact chronic pain: a framework for biopsychosocial pain research

**DOI:** 10.1097/j.pain.0000000000002951

**Published:** 2023-06-16

**Authors:** Christopher Eccleston, Emma Begley, Hollie Birkinshaw, Ernest Choy, Geert Crombez, Emma Fisher, Anna Gibby, Rachael Gooberman-Hill, Sharon Grieve, Amber Guest, Abbie Jordan, Amanda Lilywhite, Gary J. Macfarlane, Candida McCabe, John McBeth, Anthony E. Pickering, Tamar Pincus, Hannah M. Sallis, Samantha Stone, Danielle Van der Windt, Diego Vitali, Elaine Wainwright, Colin Wilkinson, Amanda C. de C Williams, Anica Zeyen, Edmund Keogh

**Affiliations:** a Centre for Pain Research, University of Bath, Bath, United Kingdom; b Department of Experimental-Clinical and Health Psychology, Ghent University, Ghent, Belgium; c Department of Psychology, The University of Helsinki, Helsinki, Finland; d School of Psychology, Aston University, Birmingham, United Kingdom; e School of Psychology, University of Southampton, Southampton, United Kingdom; f Section of Rheumatology, Cardiff University, Cardiff, United Kingdom; g Population Health Science Institute, Bristol Medical School, University of Bristol, Bristol, United Kingdom; h School of Health and Social Wellbeing, University of the West of England, Bristol, United Kingdom; i Aberdeen Centre for Arthritis and Musculoskeletal Health (Epidemiology Group), University of Aberdeen, Aberdeen, United Kingdom; j Division of Musculoskeletal and Dermatological Science, Faculty of Biology, Medicine, and Health, School of Biological Sciences, The University of Manchester, Manchester, United Kingdom; k Anaesthesia, Pain, and Critical Care Research, School of Physiology, Pharmacology, and Neuroscience, University of Bristol, Bristol, United Kingdom; l Centre for Academic Mental Health, Population Health Sciences, Bristol Medical School, University of Bristol, Bristol, United Kingdom; m Centre for Primary Care Versus Arthritis, School of Medicine, Keele University, Keele, United Kingdom; n Research Department of Clinical, Educational, and Health Psychology, University College London, London, United Kingdom; o Department of Strategy, International Business, and Entrepreneurship, School of Business and Management, Royal Holloway University of London, London, United Kingdom; p Department of Psychology, Faculty of Humanities, University of Johannesburg, Johannesburg, South Africa

## 1. Introduction

We present a framework for the study of states of chronic pain and transitions between those states. We capture in the framework the dynamic nature of pain: people live with pain that changes over time. First, we offer definitions of both acute and chronic pain and explore the contextual considerations related to the common use of this temporal dichotomy. Second, we promote the importance of incorporating the impact pain has on a person's life. Finally, we discuss the challenges and opportunities inherent in implementing this common approach. Our goal is to produce a framework for the study of the development, maintenance, and resolution of chronic pain.

Whether a single brief event or a constant feature of life, pain interrupts to prioritise protection, interferes with activity, reduces quality of life, and can alter identity.^44^ Protection is achieved by escape from harm, avoidance of perceived danger, withdrawal for respite and repair, and communication of incapacity and environmental risk; longer-term protection is achieved by learning the cues for pain and injury.^53^ From this perspective, pain is most usefully considered a need state, fundamentally a motivational drive to protect.^49^ This approach centres our attention on the consequences of pain for the person in their context, on its duration and its impact.

## 2. A person's pain status

Pain is defined as “an unpleasant sensory and emotional experience associated with, or resembling that associated with, actual or potential tissue damage.”^34^ There is a logical case of a state of “no pain,” but to have no pain is a rare occurrence, only recently made possible by the advent of anaesthesia/analgesia. A state of the continuous absence of any pain at all is profoundly abnormal, appearing only as congenital nociceptor deficiency or dysfunction. It is far from adaptive, notably leading to major clinical problems associated with the absence of defensive responding and learning with a consequent severe shortening of life expectancy.^9,50,51^

### 2.1. Acute and chronic

The terms “acute” and “chronic” represent a temporal dichotomy, with “acute” meaning short lived or immediate, and chronic meaning long term. Colloquially, however, both can be used interchangeably to mean “bad,” causing confusion in clinical encounters when patients use these as terms for severity or impact and clinicians use them for temporality.^5^

In this article, we use “acute pain” to mean pain of short duration, without any implication of severity or urgency. The pragmatic challenge in its use is the length of this short duration. Acute pain is typically defined as lasting from onset to 3 months in duration and so encompasses everything from a momentary muscular “cramp” to postoperative pain.^27,52^ Therefore, acute pain is both common and a normal part of everyday life. The few studies that establish the base rate of everyday pain generally report high incidence. For example, in 1 observational study of everyday pain in 3- to 7-year-olds, the event rate was 0.33 incidents per hour per child.^31^ Painful bumps and scrapes in the playground are normal. For adults, episodes of naturally occurring acute pain are also common. In a Europeanwide study of 8506 patients, 70% of adults reported at least 1 pain event a month (such as headache, menstrual pain, and muscle pain).^48^ Acute pain can also occur deliberately as part of a social process (eg, body adornment, contact sport, or ritual). However, most everyday acute pain does not require clinical intervention; it is self-limiting.

Clinical studies often focus on pain related to medical procedures or that occurs as the result of illness, disease, or injury (accidental, self-induced, or medically induced), and although acute pain can be relatively straightforward to manage, in particular when the timing and extent of trauma are controlled, it is not always so simple. Acute pain can be complicated for healthcare professionals to assess and manage when there are concurrent symptoms or conditions, it occurs in the presence of chronic pain, or when communication about pain is difficult, for instance in an emergency.^23^

Furthermore, we recognise that the 3-month limit is an arbitrary distinction that can lead to problems. For example, it does not account for life stage. Consider the case of new-born babies who receive repeated needle sticks for diagnostic tests: Each pain may be acute, but the pain has been present for most of the child's life. For a baby, less than 3 months is not a “short” duration. These and other concerns have led some to question the focus on duration in definitions, suggesting instead a focus on presumed mechanism.^8^

Pain of longer duration is known as “chronic pain.” Typically, chronic means having lasted for 3 months or more, as captured in the IASP definition and more recently included in *ICD-11*.^38^ Historically, chronic pain was considered to start at 6 months for adults and 3 months for children. The problem of definitions that privilege duration has been discussed,^46^ and 3 months for adult and child is now thought more clinically relevant. Given the consensus on the use of a 3-month definition, this serves as the dividing boundary that differentiates chronic from acute pain.

This definition has some difficulties. As with acute pain, 3 months is an absolute cutoff: 3 months for a 6-month-old is 50% of a life, whereas for a 60-year-old, 3 months is 0.4% of a life. If a child of 2 months has lived their life in pain, this definition of chronic would not apply, which in some circumstances would be more clinically meaningful.^25^ Furthermore, many people report pain that changes in quality or location and which fluctuates and/or is episodic. Consider that the International Headache Society classifies a number of chronic headaches as episodic with different decisions about the number, frequency, and extent of episodes,^20^ as do the Rome IV Diagnostic Criteria for Functional Gastrointestinal Disorders, which are less exact and use phrases such as “continuous,” “nearly continuous,” and “intermittent.”^37^ Including frequency in the definition of chronic low back pain may be useful and lead to a more accurate identification of treatment responders.^22^ In short, a category of chronic pain needs to encompass the experience of people who have intermittent, episodic, or continuous pain, pain of different quality and intensity, and the pain may be a sole primary complaint, secondary to disease and illness, or one of several chronic complaints.^29,43^

The variability in nosology emerging from different clinical specialities, and considerations of duration in the context of longevity, does not negate the value of a simple duration dichotomy (acute–chronic). We argue for the informed use of context when considering its use, and the need to look beyond simple labels when combining data or insights. It is a useful starting point to explore the specific features of that temporal definition in context.

### 2.2. High- and low-impact acute and chronic pain

Pain can impact multiple aspects of a person's life. We prefer “impact” over other common terms, such as disability, suffering, or distress, because it draws attention to the diverse effects of pain on the person. It is generally used conditionally to refer to the consequences of pain on a particular outcome.^13,39,42^

Duration is not a good predictor of impact. A momentary pain of an accident, incident, or procedure can have drastic effects on a person. One example is the cumulative effects of repeated exposure to acute pain in new-born babies on brain development.^2^ Another is the potential role of discrete painful events as psychologically traumatic, leading to major impact.^35^ And, a third is of uncontrolled pain near end of life, which may last less than 3 months but which can be devastating for a person and significant others.^28^

Discriminating by impact is more common in considerations of chronic pain. In particular, “high-impact chronic pain”has been defined in the United States as activity limitation^47^ and later as activity and participation limitation,^33,39^ contrasted with a category of chronic pain without limitations. This thinking was more recently captured in the Graded Chronic Pain Scale Revised as high impact compared with mild or bothersome pain.^45^ These categories allow for greater discrimination when trying to bridge between population-based studies of prevalence of chronic pain and clinical studies with adults expressing healthcare needs. The prevalence of adults with high-impact chronic pain is more typically estimated conservatively as at least 5%, in contrast to the headline population figures for all chronic pain, conservatively estimated at 20%.^33,55^

The idea that chronic pain can have low or no impact is an interesting one. Indeed, the potential for the existence of pain without impact is at the heart of the biopsychosocial model^6,16,17^ and a treatment goal in psychological rehabilitation.^54^ Although the complete resolution of chronic pain is desirable as a treatment objective, the transition to a state of low(er) impact chronic pain is often more realistic and still an important objective for individuals, healthcare providers, and society. An example is in the context of normal ageing with accommodation to life with increasingly unreachable goals achieved by altering those goals.^12^

For our purposes, high impact is defined by the extent of difficulties in function and disability (self-care, occupational engagement, and social activities)^26^ in line with the WHO. Again, we propose an informed and context-dependent use, with the need to look beyond simple labels when combining data or insights. It is a useful starting point to explore the specific features of how high impact can be determined from the available measurement.

## 3. States and transition

**Figure 1.**
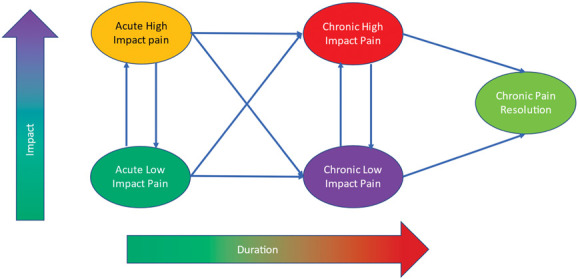
A framework for the establishment, maintenance, and adaptation of high- and low-impact chronic pain: Pain is described with 2 dominant dimensions: duration and impact. Both are dichotomised to establish specific states. Duration is split into acute pain defined as pain lasting less than 3 months and chronic as pain of 3-month duration or longer. Impact is split into low and high impact defined as a minor or major impact on self-care, occupational, and social activities. No specific measurement technology or method for determining the cutoffs is prescribed because this is a frame to capture multiple contexts of study. These 2 dimensions allow for 5 possible states represented in the oval shapes, including: acute low-impact pain and acute high-impact pain, chronic low-impact pain and chronic high-impact pain, and finally a special case of resolution of chronic pain. These 5 states allow for 10 possible transitions over time, which are given in Table 2. One cannot change from chronic to acute pain. This is a static representation of a set of dynamic processes, and we recognise that one can move between states over time, especially between chronic low impact and chronic high impact. We recognise also that chronic pain, once resolved, can relapse.

Taking duration and impact together, we propose a transitional framework for the study of 5 categorical “states” (Table 1 and Fig. 1), which include acute low-impact pain, acute high-impact pain, chronic low-impact pain, chronic high-impact pain, and a “resolved” no chronic pain state.

**Table 1:** States of pain.

Duration	Impact	Features
Acute pain	Low impact	Pain of less than 3-mo duration that is not associated with major self-care, occupational, and social activity restrictions
		Pain of less than 3-mo duration can occur in a normal everyday context. Examples are inoculation, minor injury, or pain incidental to aesthetics or recreation
Acute pain	High impact	Pain of less than 3-mo duration that is associated with major social, personal, or role restrictions
		Pain of less than 3-mo duration can have a major impact. Examples are major trauma, headache, end of life pain
Chronic pain	Low impact	Pain of 3 mo or more duration that is not associated with major social, personal or role restrictions
		Pain of 3 mo or longer can be related to a disease or be a primary disorder of the nervous system but can have minor impact
Chronic pain	High impact	Pain of 3 mo or more duration that is associated with major social, personal, or role restrictions
		Pain of 3 mo or longer can be related to a disease or be a primary disorder of the nervous system but can have major impact
Chronic pain (resolution)	No impact	The chronic pain of interest can progress to a new state of resolved chronic pain
		This new state is similar to acute pain of low impact in which everyday pain may occur but is always in the context of having had chronic pain

Although we refer here to pain duration (acute, chronic) and impact (high, low) as dichotomies, we recognise the continuous, overlapping and dynamic aspect of the pain experience. For many people, pain is an additional burden to other diseases. Our choices here are illustrative not ontological providing a framework for investigation—placing an emphasis on measurement and its use within individual investigations. In line with the US pain strategy,^26^ we recognise that introducing categories creates opportunities for research, in particular population-based research, but can under some circumstances lead to a statistical loss of information.

Table 2 outlines 10 possible trajectories of change in states, representing transitions (or absence of transition) in a person's pain state. We are interested in the onset of chronic pain, whether it is low or high impact, and its starting point of low- or high-impact acute pain. We are also interested in no change, or the maintenance of chronic pain, whether low or high impact, and the factors that lead to people becoming “stuck” in their pain state.^3^ And finally, we are interested in change in state, worsening from low impact to high impact, or improving from high impact to low impact, or a resolution from chronic low- or high-impact pain back: transition to a new normal state in which the specific pain(s) meeting the criteria for chronicity has/have resolved, but the natural rate of everyday pain resumes. These states and transitions are outlined in Figure 1.

**Table 2:** Possible transitions between chronic pain states.

Chronic pain status	1st observation	2nd observation
Onset	Acute low impact	Chronic low impact
Onset	Acute low impact	Chronic high impact
Onset	Acute high impact	Chronic low impact
Onset	Acute high impact	Chronic high impact
Change (worsening)	Chronic low impact	Chronic high impact
Change (improving)	Chronic high impact	Chronic low impact
Change (resolution)	Chronic low impact	Chronic pain resolution
Change (resolution)	Chronic high impact	Chronic pain resolution
Maintenance	Chronic low impact	Chronic low impact
Maintenance	Chronic high impact	Chronic high impact

## 4. Further considerations

Our focus on duration and impact raises several issues for consideration:

(1) Pain can be described by its pathological cause, mechanism, intensity, location, frequency, diurnality, or as a collection of features in a measure of severity. Such features are important, but, in this framework, they would be held in analyses as potential predictors, correlates, or process variables in an examination of impact and duration rather than part of their definition.
(2) The premise that a person can have chronic pain with low impact clashes with the *ICD-11* definition of primary chronic pain, a clinical diagnostic scheme that assumes high impact as a core part of the definition.^30^
(3) The concept of transition has been questioned because of an often tacit acceptance of a change in mechanism from acute to chronic pain. We make no reference to specific mechanisms but agree with the recommendation, where possible, “…to track individual pain types over time because they are likely to have different pain progression and resolution mechanisms and require different interventions.”^15^
(4) We also acknowledge that at any time point, acute and chronic pain can co-occur, but in this framework are focusing on the transition to/from chronic pain.
(5) Related is the assumption that chronic pain is pain that is refractory to treatment. There is a need to establish evidence for refractory chronic pain monitoring treatment(s) and their unsuccessful outcomes. To date, there is no broadly accepted and generalisable definition of treatment-resistant chronic pain.
(6) Similarly, Figure 1 is a static representation of changes in pain state. We recognise that one can move between high- and low-impact pain, and that pain can resolve and then relapse.^40^
(7) Living with longstanding pain can have a broad lasting impact on life that might endure past pain resolution.
(8) A focus on duration and impact privileges the individual. Chronic pain has an impact beyond the individual to other individuals, to society and to the economy.

This duration-impact framework has been developed in the context of a major UK research programme investigating the psychosocial determinants of high-impact chronic pain^7^ funded by the Advanced Pain Discovery Platform.^1^ The APDP has a focus on determining the causal influences on the onset and maintenance of high- and low-impact chronic pain. The consortium^7^ is exploring determinants of pain-state transitions using existing databases such as ALSPAC,^4,18,32^ ELSA,^14^ UK BIOBANK,^41^ HWW,^24^ and HEAF^21^ the synthesis of findings across published studies,^19^ and through new investigations.

This framework is the first step in helping to clarify clinical and research questions. First, we need to understand how to manage the uncertainty inherent in the use of measurement technology designed to capture impact and establish how far what has already been measured corresponds with, or diverts from, this framework. Second, as we are interested in factors that are causally relevant to the onset, maintenance, and change in states over time, testing causal models needs to be carefully formulated. And third, this framework can direct the selection of appropriate endpoints for intervention in attempting to alter unwanted pain states. As important as pain offset (resolution) is the improvement in impact status, from high to low. A reasonable treatment outcome for many, and therefore a clinical endpoint, is to move from high-impact to low-impact chronic pain.^10,11,36^

## 5. Conclusion

We propose a framework for studying the biopsychosocial influences on the onset, maintenance, and change in of chronic pain state. In accepting and interrogating the common dichotomies of duration (acute, chronic) and impact (high, low), we recognise the challenges inherent in dichotomizing continuous and dynamic experience. Pragmatically, however, this allows us to propose 5 unique states of pain and 10 transitions. This framework promotes a consideration of impact over time on the person with pain and will enable investigation of the causal determinants of states and changes in state.

## Conflict of interest statement

The authors have no conflict of interest to declare.
